# Omega-3 Fatty Acids DHA and EPA Reduce Bortezomib Resistance in Multiple Myeloma Cells by Promoting Glutathione Degradation

**DOI:** 10.3390/cells10092287

**Published:** 2021-09-02

**Authors:** Jing Chen, Esther A. Zaal, Celia R. Berkers, Rob Ruijtenbeek, Johan Garssen, Frank A. Redegeld

**Affiliations:** 1 Division of Pharmacology, Utrecht Institute for Pharmaceutical Sciences, Faculty of Science, Utrecht University, 3508 TB Utrecht, The Netherlands; j.chen@uu.nl (J.C.); j.garssen@uu.nl (J.G.); 2 Biomolecular Mass Spectrometry and Proteomics, Bijvoet Center for Biomolecular Research, Utrecht Institute of Pharmaceutical Sciences, Utrecht University, 3508 TB Utrecht, The Netherlands; e.a.zaal@uu.nl (E.A.Z.); c.r.berkers@uu.nl (C.R.B.); 3 Division of Cell Biology, Cancer & Metabolism, Department of Biomolecular Health Sciences, Faculty of Veterinary Medicine, Utrecht University, 3508 TD Utrecht, The Netherlands; 4 Pamgene International, 5200 BJ ‘s-Hertogenbosch, The Netherlands; rru@genmab.com; 5 Nutricia Research, 3508 TC Utrecht, The Netherlands

**Keywords:** multiple myeloma, bortezomib, omega-3 fatty acids, DHA, EPA, drug resistance, transcriptome, metabolome

## Abstract

Multiple myeloma (MM) is a hematological malignancy that exhibits aberrantly high levels of proteasome activity. While treatment with the proteasome inhibitor bortezomib substantially increases overall survival of MM patients, acquired drug resistance remains the main challenge for MM treatment. Using a combination treatment of docosahexaenoic acid (DHA) or eicosapentaenoic acid (EPA) and bortezomib, it was demonstrated previously that pretreatment with DHA/EPA significantly increased bortezomib chemosensitivity in MM cells. In the current study, both transcriptome and metabolome analysis were performed to comprehensively evaluate the underlying mechanism. It was demonstrated that pretreating MM cells with DHA/EPA before bortezomib potently decreased the cellular glutathione (GSH) level and altered the expression of the related metabolites and key enzymes in GSH metabolism, whereas simultaneous treatment only showed minor effects on these factors, thereby suggesting the critical role of GSH degradation in overcoming bortezomib resistance in MM cells. Moreover, RNA-seq results revealed that the nuclear factor erythroid 2-related factor 2 (NRF2)-activating transcription factor 3/4 (ATF3/4)-ChaC glutathione specific gamma-glutamylcyclotransferase 1 (CHAC1) signaling pathway may be implicated as the central player in the GSH degradation. Pathways of necroptosis, ferroptosis, p53, NRF2, ATF4, WNT, MAPK, NF-κB, EGFR, and ERK may be connected to the tumor suppressive effect caused by pretreatment of DHA/EPA prior to bortezomib. Collectively, this work implicates GSH degradation as a potential therapeutic target in MM and provides novel mechanistic insights into its significant role in combating bortezomib resistance.

## 1. Introduction

MM, the second most prevalent hematological malignancy, is charactered by the clonal proliferation of antibody-secreting plasma cells in the bone marrow [1]. Given that MM cells inevitably produce large amounts of misfolded or unfolded proteins, proteasome inhibitor-based drugs are widely used as first line therapy for MM [2]. Additionally, inhibiting proteasome activity has been shown to protect IκBα (inhibitor of NF-κB) from degradation, thereby tilting the signaling balance in these cells toward apoptosis [3]. Since bortezomib (Velcade/PS-341), a boronic acid dipeptide that reversibly binds to the β2 and β5 subunits in the 20S core of the proteasome, was approved for the treatment of patients with relapsed/refractory or newly diagnosed MM [4], it has substantially increased overall survival of MM patients over the past decade. However, most patients invariably relapse after an effective initial treatment or become refractory, mainly due to the development of drug resistance [5]. Interestingly, recently, daratumumab, a CD38-targeting human monoclonal antibody, in combination with bortezomib, has been approved by the FDA for relapsed/refractory MM patients after bortezomib-based therapy [6]. However, it remains a highly unmet need for developing novel drugs or combination therapeutic strategies to increase chemosensitivity for MM treatment.

Intensive studies have focused on understanding the molecular mechanisms involved in bortezomib-associated resistance. Upregulation of ubiquitin–proteasome activity has been frequently observed in bortezomib-resistant MM cells, which was mostly attributed to the elevated expression of the proteasomal β catalytic subunits or site mutation(s) in the bortezomib binding pocket [7]. Another key mechanism of the resistance is the aberrant activation of cellular signaling pathways. In addition to proteasomal degradation, unfolded/misfolded proteins also aggregate into aggresome and subsequently undergo autophagy-mediated degradation. Significantly increased activity of the aggresome-autophagy pathway has also been detected in proteasome inhibitors-resistant MM cells [8]. Furthermore, constitutive activation of pro-survival signaling pathways, such as NF-κB, EGFR/JAK2/STAT3 and PI3K/AKT, has been reported to decrease bortezomib chemosensitivity in MM cells [9]. Moreover, NRF2, a transcription factor that directly regulates the expression of a set of genes involved in antioxidation and cytoprotection, was found to be positively correlated with bortezomib-induced resistance [10]. Additionally, downregulation or loss-of-function mutation of tumor suppressors (e.g., PTEN and p53) have been implicated in the development of drug resistance during bortezomib-based chemotherapy [11,12]. Interestingly, cancer cells often rewire their cellular metabolism to produce sufficient energy and essential materials to support cell proliferation, survival, invasion, metastasis, and chemoresistance. Recent studies have demonstrated that the pentose phosphate pathway (PPP), the serine synthesis pathway (SSP), and glycolysis were raised in patients with relapsed/refractory MM after bortezomib-based therapy [13,14], indicating the critical role of cellular metabolism in the acquisition of resistance.

In our previous work, omega-3 polyunsaturated fatty acids (PUFAs), i.e., DHA and EPA, were shown to have selective cytotoxicity against primary MM cells and MM cell lines, but not in normal human PBMCs [15,16]. Moreover, we recently reported EPA and DHA induced apoptosis in human MM cell lines, including L363, OPM2, MM.1S, and U266, through activating intrinsic (mitochondrial) and extrinsic (death receptor) apoptotic pathways. More importantly, pretreating MM cells with DHA/EPA prior to bortezomib synergistically enhanced the efficiency of bortezomib in inducing apoptosis, suggesting their roles in overcoming chemoresistance in this case, whereas simultaneous treatment with bortezomib and DHA/EPA decreased bortezomib chemosensitivity [17]. These findings indicated that the order for the combinational use of DHA/EPA with bortezomib can exert completely opposite anti-myeloma effects and promoted us to explore the underlying mechanisms. In this study, transcriptome and metabolome analyses were performed in MM cells, which indicated that pretreating MM cells with DHA/EPA before bortezomib potently decreased the cellular GSH level and altered the expression of the key enzymes in GSH metabolism, whereas simultaneous treatment only showed moderate effect on these factors. Moreover, CHAC1 was regulated possibly by activating the NRF2-ATF3/4 pathway, which may also contribute to the decrease in the GSH in pretreated cells. These novel results report the clues for explaining the synergistical effect that we observed before and provide a molecular basis for overcoming bortezomib resistance in MM.

## 2. Materials and Methods

### 2.1. Reagents

DHA (D2534) and EPA (E2011) were purchased from Sigma and dissolved in ethanol to produce a 100 mM stock solution. Bortezomib was purchased from LC laboratories and dissolved in DMSO. An Annexin V apoptosis detection kit (88-8005-74; Carlsbad, CA, USA) was obtained from Thermo Fisher.

### 2.2. Cell Culture

The OPM2 cell line was cultured in RPMI-1640 medium supplemented with 10% fetal calf serum (FCS), 2 mM of L-glutamine, 100 IU of penicillin, and 100 mg/mL of streptomycin. The bortezomib-resistant cell line, RPMI8226-BTZ/100, was maintained in the same medium as described above with the addition of 100 nM bortezomib.

### 2.3. Cell Death Analysis by Flow Cytometry

Treated MM cells were washed with cold PBS and binding buffer. Cells were resuspended in 100 mL of binding buffer and stained with 5 µL of Annexin V for 15 min at room temperature in dark. Then, cells were washed with binding buffer and resuspended with 100 µL of binding buffer containing 2.5 µL of PI. The percent of dead cells (Annexin V+) cells was determined by the flow cytometry (BD FACSCanto II, San Jose, CA, USA).

### 2.4. RNA-Seq Differential Gene Expression Analysis

OPM2 cells were seeded in a 12-well plate at density of 10 × 103 cells/well and incubated with the indicated concentrations of compounds. Total RNA was extracted from cells using RNeasy Mini Kit (74104, Qiagen, Hilden, Germany) and RNA integrity was determined by Agilent 2100 analysis. RNA integrity numbers (RIN) of all samples were greater than 8.0. RNA-seq libraries were prepared and sequenced at Novogene (Novogene, Cambridge, UK). Data were analyzed using Novosmart software (Novogene, Cambridge, UK). Kyoto Encyclopedia of Genes and Genomes (KEGG) (Novosmart; Cambridge, UK) and Reactome (www.reactome.org) were used to perform enrichment analysis of the DEGs.

### 2.5. Metabolomic Analysis

Treated OPM2 and RPMI8226-BTZ/100 cells were washed twice with ice-cold PBS and then lysed with a lysis buffer (40% acetonitrile, 40% methanol and 20% MQ water) by quick vortexing at 4 °C. Cell lysates were collected by centrifugation at 4 °C for 15 min at 13,000 rpm and subsequently subjected to LC-MS-based metabolomics analysis to determine the metabolome changes [12]. MetaboAnalyst (version 5.0; Xia Lab, Montreal, QC, Canada) was used for pathway enrichment analysis of the selected metabolites.

### 2.6. Statistics

Statistical analyses were performed using GraphPad Prism software (version 9.0; San Diego, CA, USA). Statistical significance between the tested groups was determined using one-way ANOVA. *p* < 0.05 was considered significant.

## 3. Results

### 3.1. Differential Changes in Transcriptome upon Treatment with DHA, EPA, or Bortezomib in MM Cells

**Figure 1:**
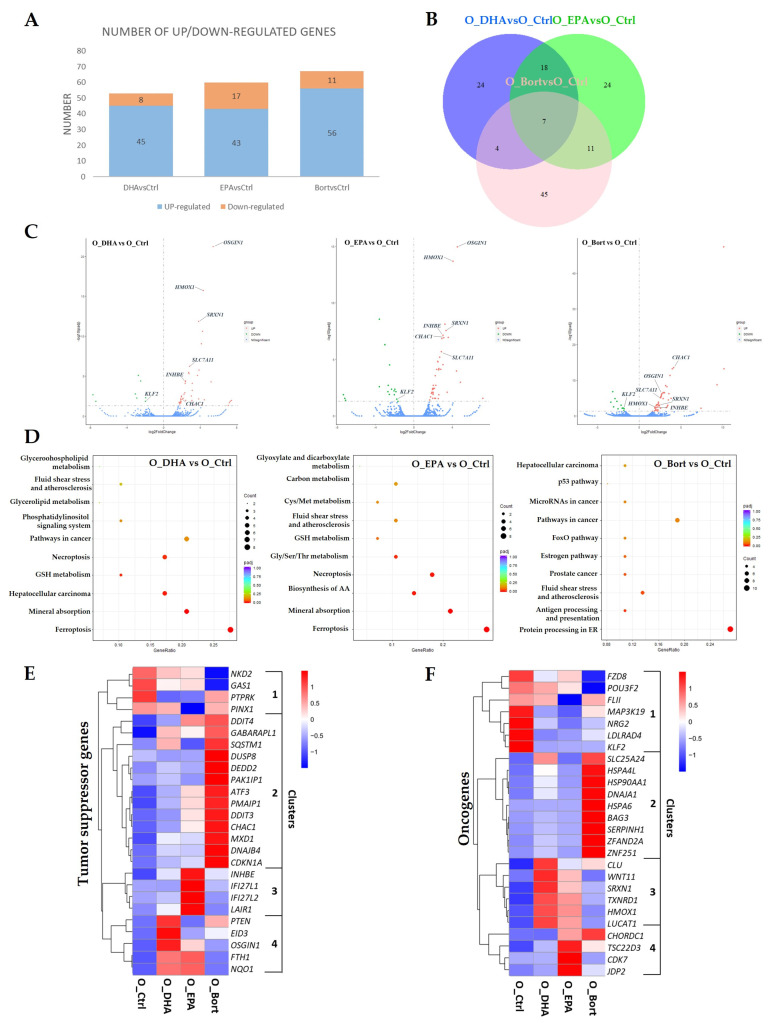
Transcriptomic profiling in response to DHA, EPA, or bortezomib in the OPM2 cell line. Cells were treated with 50 µM of DHA/EPA (6 h) or 10 nM of bortezomib (4 h), and then RNA-seq analysis were performed. (**A**) Number of significantly up- and down-regulated DEGs identified in different comparison groups. *p*_adj_ < 0.05. (**B**) Venn diagrams showing the numbers of overlapping and non-overlapping DEGs in three comparison groups. (**C**) Volcano plots summarizing the DEGs upon treatment with DHA (left), EPA (middle) or bortezomib (right). The seven overlapping DEGs from (**B**) were highlighted. Green, downregulated DEGs; red, upregulated DEGs. (**D**) KEGG pathway analysis of DEGs in treated cells compared to the control. The counts present the number of DEGs enriched in a particular pathway. Different colors represent *p*_adj_ values. Hierarchical clustering heatmaps depicting the levels of differentially expressed tumor suppressor genes (**E**) and oncogenes (**F**) from RNA-Seq analysis of the control and DHA/EPA/bortezomib-treated cells.

It was reported previously that DHA and EPA can induce apoptosis in MM primary cells [15] and MM cell lines [17]. To further investigate the underlying molecular mechanisms, RNA-seq analysis in OPM2 cells was performed and the differentially expressed genes (DEGs) between treated and untreated cells were identified (*p*_adj_ < 0.05, |log_2_ fold change| > 2) (Figure 1A). The Venn diagram showed that seven common genes were differentially expressed after treatment with DHA, EPA, or bortezomib (Figure 1B), which were represented in the volcano plots (Figure 1C). Among those common genes, *KLF2*, a transcription factor that has been recently shown to be essential for MM cell survival [18], was significantly decreased by all treatment. The other four NRF2-regulated genes (*HMOX1*, *OSGIN1*, *SLC7A11*, and *SRXN1*) [19] were consistently increased, indicating that these cell death inducers might activate NRF2-associated signaling pathways in OPM2 cells. Besides, two pro-apoptotic genes (*CHAC1* and *INHBE*) [20,21] were also upregulated in treated cells.

To further identify the potential signaling pathways associated with above DEGs, Kyoto Encyclopedia of Genes and Genomes (KEGG) pathway enrichment analysis was performed (*p*_adj_ < 0.05, |log2 fold change| > 2). It showed that the DEGs regulated by DHA/EPA were mainly enriched for genes involved in ferroptosis, mineral absorption, glutathione metabolism, necroptosis, and metabolism of amino acids (Figure 1D, left and middle panels), suggesting that ferroptosis and necroptosis might be associated with DHA/EPA-induced cell death in MM cells. Additionally, the DEGs regulated by bortezomib were only significantly enriched for protein processing in endoplasmic reticulum and antigen processing and presentation (Figure 1D, right panel).

To obtain a comprehensive understanding of the molecular mechanisms underlying their anticancer activities, the expression levels of all tumor suppressor genes and oncogenes included in the DEGs were further examined. The expression levels of genes with similar patterns were clustered together in the heatmaps (Figure 1E,F). A total of 26 tumor suppressor genes were assigned into four clusters, of which one (cluster 2) encompasses 13 bortezomib-upregulated genes, and cluster 3 and 4 mainly represent DHA/EPA-upregulated genes. Cluster 1 contains several downregulated tumor suppressor genes, including *NKD2*, *GAS1*, *PINX1*, and *PTPRK* [22,23,24,25]. Next, pathway enrichment analysis of the genes in each cluster using the Reactome pathway tool was performed. The results showed that the genes regulated by bortezomib in cluster 2 were mostly involved in response of EIF2AK1 (HRI) to heme deficiency, cellular responses to stress and external stimuli, the p53 signaling pathway, p21-mediated cell cycle arrest, and the ATF4 signaling pathway, whereas the genes regulated by fatty acids in cluster 3 and 4 were mainly enriched in the p53 signaling pathway, PTEN regulation and transcription, iron uptake and transport (Table S1). Similarly, 26 oncogenes derived from the DEGs were also assigned into four clusters, but only 7 genes in cluster 1 were expectedly downregulated. Among these genes, *FZD8*, a receptor for activating canonical WNT/β-catenin signaling [26], and *POU3F2*, a target gene of the WNT/β-catenin pathway that has shown tumorigenic effect in cancer [27], were significantly downregulated in bortezomib-treated cells, suggesting that the WNT/β-catenin pathway might be an important target pathway by bortezomib in MM cells. Elevated expression of *NRG2* (a ligand of HER/ERBB receptor for activating the EGFR pathway) was found to be correlated with increased tumor aggressiveness [28]. The decreased *NRG2* expression by DHA/EPA and bortezomib suggested the involvement of the EGFR signaling pathway in their cytotoxicity in MM cells. Oncogene, *LDLRAD4*, which was highly expressed in multiple cancers (e.g., hepatic cancer, breast cancer and colon cancer) [29], and *KLF2*, an essential transcriptional factor for MM cell survival [18], were also downregulated in all treated cells. *FLII* and *MAP3K19* were downregulated by fatty acids DHA/EPA. *FLII* has been recently reported to promote breast cancer progression via inhibiting p62-mediated selective autophagy [30] and block apoptosis in colon cancer cells by regulating Ca^2+^ homeostasis [31]. *MAP3K19* has been shown to activate pro-survival pathways ERK and JNK through directly phosphorylation of MEK1/2 and MKK7 [32]. Thus, the downregulation of *MAP3K19* by DHA and EPA suggested that these fatty acids might show inhibitory effect on ERK and JNK signaling pathways.

Taken together, the molecular actions relevant for bortezomib induced cell death of MM cells might include blocking pro-survival pathways of WNT/β-catenin and EGFR and activating p53-mediated cell cycle arrest and apoptosis; however, in the case of fatty acids, DHA/EPA might inhibit ERK, JNK, and EGFR signaling pathways and activate PTEN and p53 signaling pathways, autophagy, ferroptosis, and necroptosis.

### 3.2. Differential Changes in Transcriptome upon Simultaneous Treatment with Bortezomib and DHA or EPA in MM Cells

**Figure 2:**
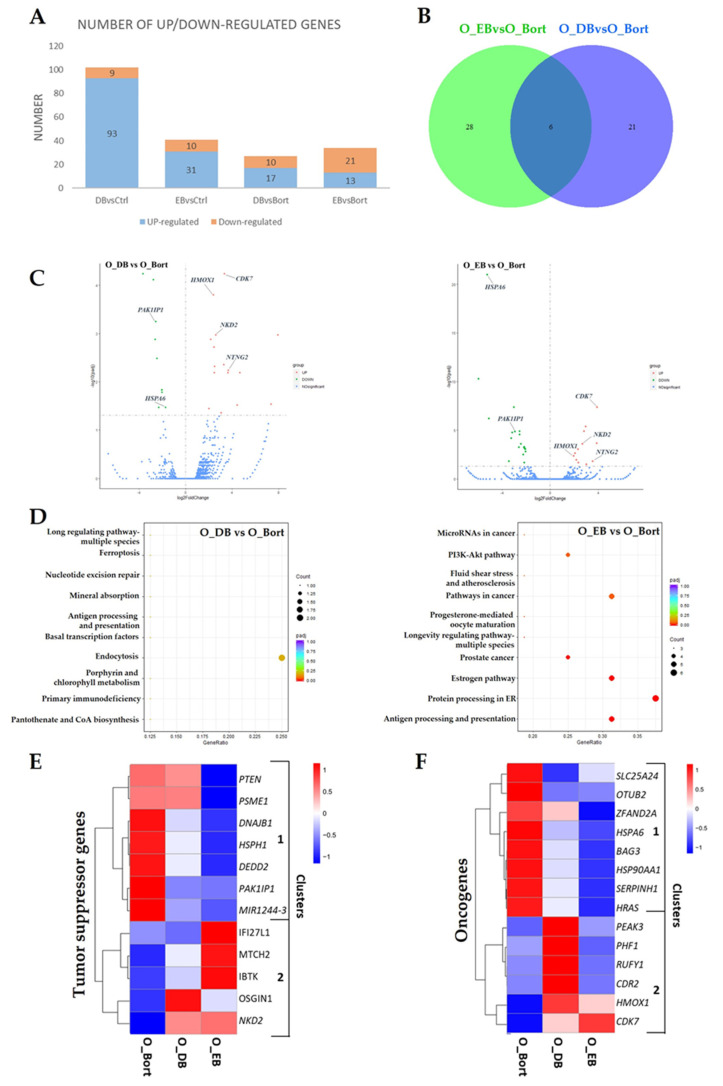
Simultaneous treatment with bortezomib and DHA or EPA altered tumor-associated gene expression in the OPM2 cell line. Cells were treated with 10 nM of bortezomib for 4 h in the presence of DHA/EPA (50 µM), and then RNA-seq analysis were performed. (**A**) Number of significantly up- and down-regulated DEGs identified in different comparison groups. *p*_adj_ < 0.05. DB, DHA, and bortezomib; EB, EPA, and bortezomib. (**B**) Venn diagrams showing the numbers of overlapping and non-overlapping DEGs in two comparison groups. (**C**) Volcano plots summarizing the DEGs upon treatment with bortezomib and DHA (left panel) or EPA (right panel). The six overlapping DEGs from (**B**) were highlighted. Green, downregulated DEGs; red, upregulated DEGs. (**D**) KEGG pathway analysis of DEGs in treated cells compared to the control. The counts present the number of DEGs enriched in a particular pathway. Different colors represent *p*_adj_ value. Left, DB vs Bort; right, EB vs Bort. Hierarchical clustering heatmaps depicting the levels of differentially expressed tumor suppressor genes (**E**) and oncogenes (**F**) from RNA-Seq analysis of bortezomib-, DB-, and EB-treated cells.

Previous results showed that simultaneous treatment with bortezomib and DHA or EPA decreased bortezomib efficacy in MM cells [17], which suggests that these fatty acids exhibit oncogenic effects when they are used simultaneously with bortezomib. RNA-seq differential gene expression analysis was performed to investigate the potential molecular mechanisms (Figure 2A). The Venn diagram and volcano plots showed that six genes were regulated by all treatment (Figure 2B,C). Of these genes, *HSPA6* and *PAK1IP1* were downregulated and *CDK7*, *HMOX1*, *NKD2*, and *NTNG2* were upregulated. *HSPA6* (also known as *HSP70B’*) has been found to stabilize anti-apoptotic proteins (e.g., Bcl-xL) through direct binding [33], thereby suggesting its oncogenic role in cancer cells. Interestingly, both overexpression and knockdown of *PAK1IP1* were found to induce p53-dependent cell cycle arrest in various cancer cell lines [34]. The upregulated overlapping genes include three oncogenes (*CDK7*, *HMOX1*, and *NTNG2*) and one tumor suppressor (*NKD2*). The upregulation of *CDK7*, a cyclin-dependent kinase that plays an important role in cell cycle progression [35], indicated the increased proliferative activity by simultaneous treatment. *NTNG2* may be considered as a potential diagnostic biomarker and therapeutic target in cancers [36]. *NKD2* is a negative regulator of the WNT signaling pathway, and its overexpression inhibited cancer cell proliferation, migration, and invasion [22].

Further KEGG pathway analysis revealed that the DEGs in EPA and bortezomib-treated cells were mostly enriched in antigen processing and presentation, protein processing in endoplasmic reticulum, and the estrogen signaling pathway, while no pathways were significantly enriched in DHA and bortezomib-treated cells. The estrogen signaling pathway has been strongly implicated in tumorigenesis, either directly or indirectly via myeloid-derived suppressor cells (MDSCs), which represent one of the major cellular populations in MM bone marrow [37]. Notably, the DEGs-associated with the estrogen signaling pathway, including several heat shock proteins (*HSPA6*, *HSPA7*, *HSP90AA2P*, and *HSP90AA1*) and the oncogene, *HRAS,* were all downregulated, suggesting that simultaneous treatment with EPA and bortezomib might inhibit the estrogen signaling pathway in MM cells.

Additionally, to investigate the molecular mechanisms underlying the oncogenic effect, the expression levels of all tumor suppressors and oncogenes-derived from the DEGs were analyzed and compared. The heatmap showed that 12 tumor suppressors formed into two clusters: cluster 1 contains seven downregulated tumor suppressors including *PTEN*, *PSME1*, *DNAJB1*, *HSPH1*, *DEDD2*, *PAK1IP1*, and *MIR1244-3*; and cluster 2 contains five upregulated tumor suppressors (*OSGIN1*, *IFI27L1*, *MTCH2*, *NKD2*, and *IBTK*) (Figure 2E). The top five genes in cluster 1 were mainly enriched for cellular response to heat shock and stress, HSF1-dependent transactivation, and activation and regulation of regulation of PTEN signaling (Table S1). The downregulation of MIR1244 was reported to contribute to cisplatin resistance in NSCLC through blocking the p53 signaling pathway [38]. In addition, 14 oncogenes were identified from the DEGs and formed into two clusters (Figure 2F). Among these genes, six were upregulated, of which *PEAK3*, *PHF1*, *RUFY1*, and *CDR2* were upregulated by DHA and bortezomib treatment, and *HMOX1* and *CDK7* were upregulated by both treatments. *PEAK3*, a member of the new kinase family 3 (NKF3) mediating cell motility and tumor progression, has been identified as a therapeutic target for acute myeloid leukemia (AML) treatment [39]. PHF1, RUFY1, and CDR2 are highly expressed in some cancers, positively correlated with tumor progression [40,41,42]. CDK7 is required to maintain the activity of CDK4 and CDK6 for cell cycle progression [35]. 

Together, these results suggested that simultaneous treatment with DHA/EPA and bortezomib reduced bortezomib chemosensitivity possibly through activating CDK7-regulated cell cycle progression and inhibiting PTEN and p53 pathways in MM cells.

### 3.3. Differential Changes in Transcriptome upon Pretreatment with DHA or EPA before Bortezomib in MM Cells

**Figure 3:**
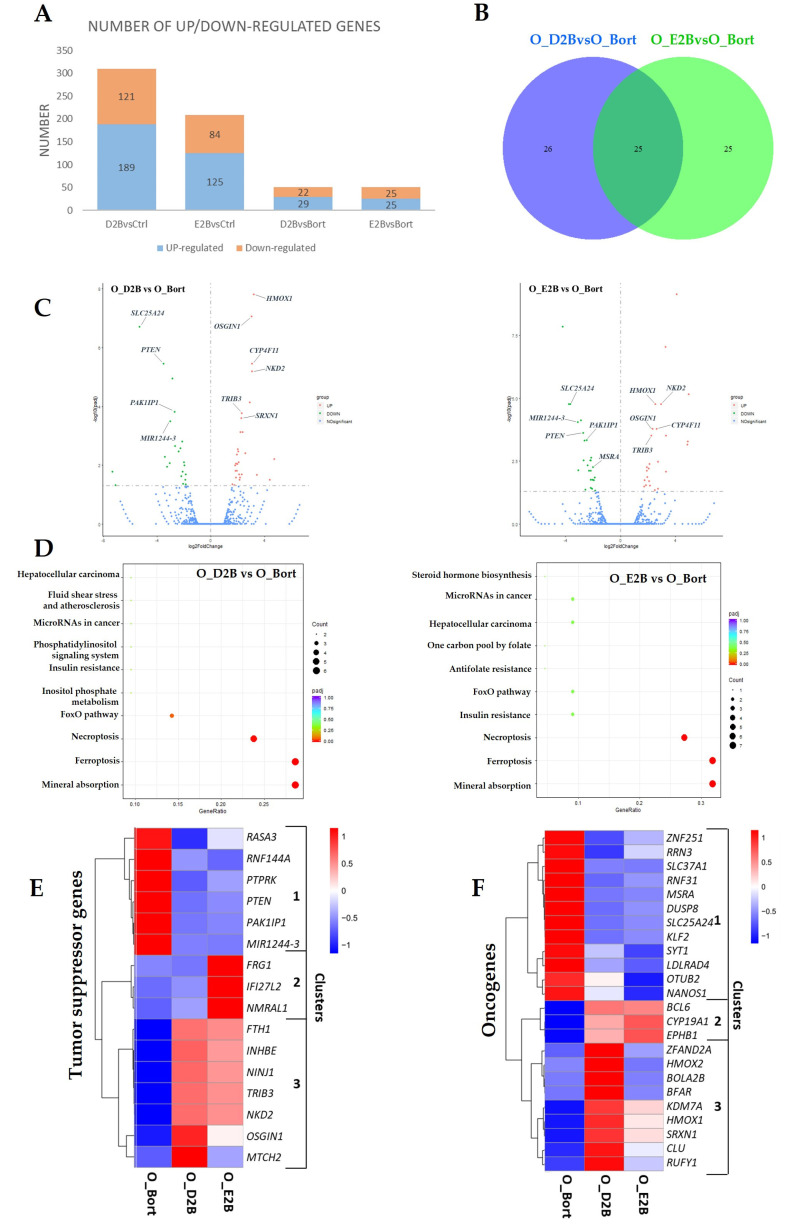
Pretreatment with DHA or EPA before bortezomib differentially regulated tumor-associated gene expression in the OPM2 cell line. Cells were pretreated with 50 µM of DHA or EPA for 0 and 2 h and treated with bortezomib (10 nM) for 4 h. Then, RNA-seq analysis was performed. (**A**) The number of significantly up- and down-regulated DEGs identified in different comparison groups. *p*_adj_ < 0.05. D2B/E2B, 2 h pretreatment with DHA/EPA plus 4 h treatment of bortezomib. (**B**) Venn diagrams showing the numbers of overlapping and non-overlapping DEGs in two comparison groups. (**C**) Volcano plots summarizing the DEGs upon treatment with D2B (left panel) or E2B (right panel). The top 10 overlapping DEGs from (**B**) were highlighted. Green, downregulated DEGs; red, upregulated DEGs. (**D**) KEGG pathway analysis of DEGs in treated cells compared to the control. The count presented the number of DEGs enriched in a particular pathway. Different colors represent *p*_adj_ value. Left, D2B vs Bort; right, E2B vs Bort. Hierarchical clustering heatmaps depicting the levels of differentially expressed tumor suppressor genes (**E**) and oncogenes (**F**) from RNA-Seq analysis of bortezomib, D2B and E2B-treated cells.

Importantly, pretreatment with DHA or EPA before bortezomib results in synergistic toxicity in MM cells [17], indicating that these fatty acids exhibit tumor suppressive effects when they are used before bortezomib. Similarly, the differently expressed genes were identified from DHA/EPA-pretreated cells compared to only bortezomib-treated cells (Figure 3A). The Venn diagram showed that 21 genes (excluding 4 pseudogenes) were regulated by all treatments (Figure 3B), of which 10 genes were downregulated and 11 genes were upregulated. The top 10 most significantly differentially expressed overlapping genes were indicated in the volcano plots in Figure 3C, including three upregulated tumor suppressors (*OSGIN1*, *NKD2* and *TRIB3*) and two downregulated oncogenes (*SLC25A24* and *MSRA*).

Furthermore, the KEGG pathway analysis revealed that the DEGs regulated by DHA/EPA pretreatment are significantly enriched for pathways associated with mineral absorption, ferroptosis, and necroptosis (Figure 3D). This suggested that pretreatment with DHA/EPA in MM cells might exert their pro-chemotherapeutic effect of bortezomib through regulating necroptosis and ferroptosis.

In addition, 16 tumor suppressors and 24 oncogenes-screened from the DEGs were segregated into three clusters, respectively. As shown in Figure 3E, cluster 1 represented six downregulated genes by DHA and EPA pretreatment; cluster 2 contained three upregulated genes (*FRG1*, *IFI27L2* and *NMRAL1*) by EPA pretreatment; cluster 3 contained the increased expression of NRF2-regulated genes (*FTH1, TRIB3*, and *OSGIN1*) [19], an ATF4 target gene (*INHBE*) [21], a negative regulator of the WNT pathway (*NKD2*) [22], a pro-apoptotic gene (*MTCH2)* [43], and a p53 target gene (*NINJ1)* [44]. Reduced expression of *FRG1* has been frequently observed in prostate cancer tissue, which contributes to tumor progression through the p38-MAPK pathway [45]. *IFI27L2* was found to play a critical role in apoptosis induction [46]. *NMRAL1* inhibits NF-κB through directly binding to IKKβ [47], suggesting its negative effect on tumorigenesis. Furthermore, downregulated oncogenes in cluster 1 (Figure 3F) might be associated with several cancer pathways, such as MAPK, TNFR1-induced NF-κB, and EGFR (Table S2).

Altogether, the above results revealed that cellular pathways of necroptosis, ferroptosis, p53, NRF2, ATF4, WNT, p38-MAPK, NF-κB, and EGFR might be involved in bortezomib chemosensitivity in MM cells increased by DHA/EPA pretreatment.

### 3.4. Pretreatment with DHA or EPA before Bortezomib Activates GSH Degradation in MM Cells

**Figure 4:**
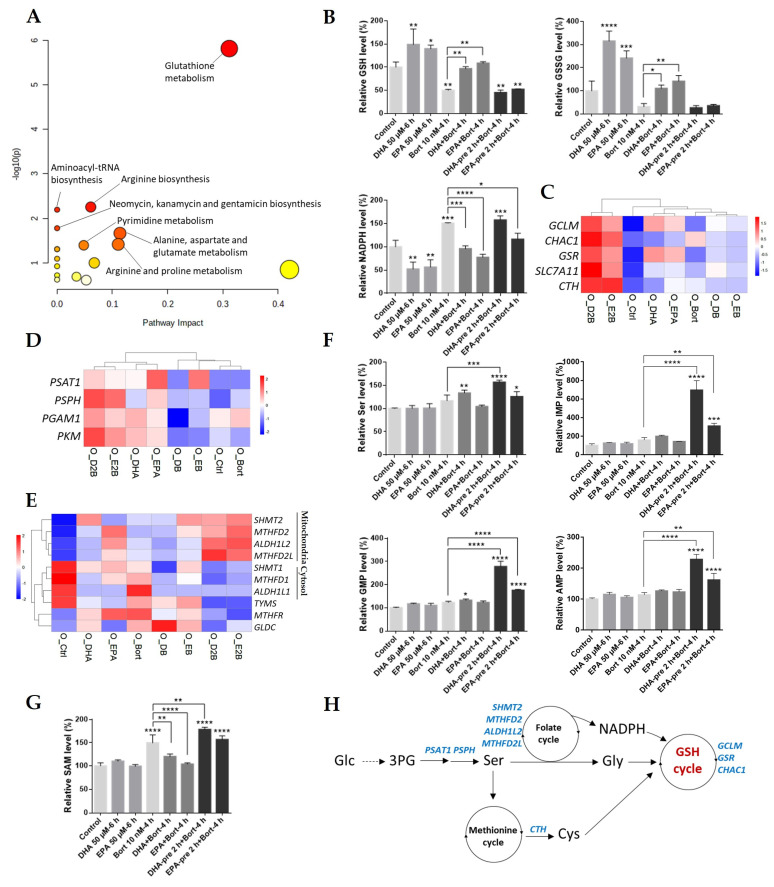
Transcriptomic and metabolomic analysis in the OPM2 cell line reveals the crucial role of GSH metabolism in increasing bortezomib chemosensitivity in MM. For metabolomic analysis, OPM2 cells were pretreated with 50 µM of DHA/EPA for 0 or 2 h, then bortezomib (10 nM) was added for 6 h treatment. (**A**) Metabolome pathway enrichment of 16 differentially regulated metabolites using MetaboAnalyst 5.0. The node color represents the *p* values, and the node size represents the pathway impact values. (**B**) The levels of GSH, oxidized GSH (GSSG) and NADPH upon different treatment. Heatmap analysis of the expression of the key enzymes involved in GSH metabolism (**C**), serine synthesis and metabolism (**D**) and folate cycle (**E**) in different conditions. (**F**) and (**G**) The levels of metabolites related to folate cycle and methionine cycle in different conditions. Data are presented as mean ± SD of three independent treatment. * *p* < 0.05, ** *p* < 0.01, *** *p* < 0.001, **** *p* < 0.0001when compared with the control. (**H**) Graphical representation of the metabolic enzymes and pathways associated with the GSH cycle in OPM2 cells. Enzymes were highlighted in blue.

To determine the changes of metabolome activity between different treatment, metabolomics analysis was performed in OPM2 cells. A total of 110 metabolites were identified based on their accurate mass and used for further differential expression analysis. Among these metabolites, 16 were differentially expressed in pretreatment as compared with simultaneous treatment (Table S4). Pathway enrichment analysis with these metabolites revealed that glutathione metabolism was most significantly enriched (enrichment *p* value = 1.55 × 10^−6^) (Figure 4A). Glutathione (GSH) is a tripeptide consisting of glutamate, cysteine, and glycine that functions to maintain cellular redox homeostasis [48]. Individual metabolites analysis showed that pretreatment with DHA/EPA significantly decreased GSH and oxidized GSH (glutathione disulfide, GSSG), whereas simultaneous treatment had no effect compared to the control (Figure 4B). It is notable that DHA/EPA markedly increased GSH and GSSG. Furthermore, *GCLM* and *GSR*, the key enzymes for GSH synthesis, were upregulated at the transcript level by single treatment with DHA/EPA or pretreatment, suggesting the activation of GSH synthesis in response to oxidative stress (Figure 4C). Interestingly, *CHAC1*, an enzyme that can be induced by oxidative stress and functions to hydrolyze GSH to Cys-Gly and 5-oxoproline [20], was upregulated by bortezomib treatment and pretreatment, further explaining the decrease in GSH levels in these cells, but not in DHA/EPA or simultaneously treated cells (Figure 4C). Moreover, the upregulation of enzyme of cysteine synthesis (*CTH*) and cystine/glutamate antiporter gene (*SLC7A11*) (Figure 4C) in pretreated cells suggested a higher demand of cysteine in these cells, which might contribute to the GSH synthesis. In addition, NADPH, a well-known cofactor with a critical role in reducing GSSG to GSH for cellular redox maintenance, was also increased in pretreated cells (Figure 4B). Together, these results indicated that DHA/EPA pretreatment induced depletion of GSH levels in OPM2 cells.

Because serine is an essential precursor for the biosynthesis of glycine and cysteine, the expression level of serine synthesis genes was analyzed. The increase in serine (Figure 4F) and upregulation of the key enzymes for serine synthesis and metabolism (*PSAT1*, *PSPH*, *PGAM1*, and *PKM*) [49] in pretreated cells (Figure 4D) indicated the activation of the serine synthesis pathway (SSP) after pretreatment.

Additionally, serine can be converted into glycine by serine hydroxymethyltransferase 2 (SHMT2) through the mitochondrial folate cycle, which is essential for de novo biosynthesis of purine and pyrimidine [49]. Thus, the expression of the folate metabolism-associated genes was further analyzed (Figure 4E). Interestingly, all genes of the key enzymes that function in the mitochondria, including *SHMT2*, *MTHFD2*, *ALDH1L2*, and *MTHFD2L*, were significantly upregulated by pretreatment compared to bortezomib treatment, whereas cytosolic folate cycle-associated genes (*SHMT1*, *MTHFD1*, and *ALDH1L1*) were all downregulated. In line with these results, we also observed increases of purines AMP and GMP, as well as their precursor IMP in pretreated cells (Figure 4F), suggesting the higher activity of purine synthesis by DHA/EPA pretreatment. These results demonstrated that the mitochondrial folate cycle was activated in OPM2 cells by pretreating with DHA/EPA.

The methionine cycle is tightly coupled to folate cycle, which generates homocysteine, a precursor of cysteine, for GSH synthesis [49]. Our result showed that S-adenosyl-methionine (SAM), a major methyl donor for most cellular methylation reactions that produce S-adenosylhomocysteine (SAH) as a by-product for homocysteine synthesis, was substantially increased by pretreatment but was decreased by simultaneous treatment compared to bortezomib treatment (Figure 4G), indirectly indicating the activation of methionine metabolism by DHA/EPA pretreatment in OPM2 cells.

Overall, these results demonstrated that pretreatment with DHA/EPA activated GSH metabolism through activation of the SSP pathway, the mitochondrial folate cycle, methionine cycle-associated GSH synthesis, and CHAC1-mediated GSH degradation in OPM2 cells, resulting in depletion of the GSH pool (Figure 4H).

### 3.5. Pretreatment with DHA or EPA before Bortezomib Activates GSH Degradation in the Bortezomib-Resistant MM Cell Line RPMI8226-BTZ/100

**Figure 5:**
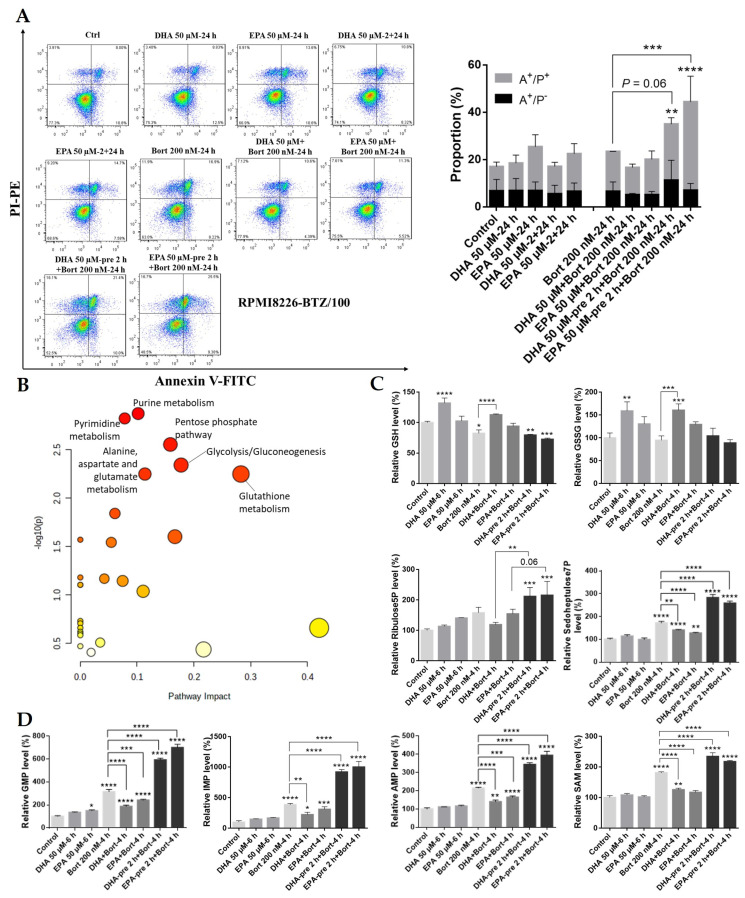
GSH metabolism plays a critical role in increasing bortezomib chemosensitivity in the bortezomib-resistant MM cell line. (**A**) RPMI8226-BTZ/100 was pretreated with 50 µM of DHA or EPA for 0 or 2 h and then incubated with bortezomib (200 nM) for 24 h. Apoptotic cells were determined by Annexin-V and PI staining. (**B**) Metabolome pathway enrichment of 24 differentially regulated metabolites using MetaboAnalyst 5.0. The node color represents the *p* values, and the node size represents the pathway impact values. (**C**) and (**D**) The levels of the indicated metabolites upon different treatment. Data are presented as mean ± SD of three independent treatment. * *p* < 0.05, ** *p* < 0.01, *** *p* < 0.001, **** *p* < 0.0001 when compared with the control.

The results, as mentioned above, suggest that targeting GSH metabolism may be a useful strategy to combat bortezomib resistance in MM, in line with previous studies [13,50,51]. Notably, the synergetic effects of DHA/EPA on bortezomib efficacy were observed as well in the bortezomib-resistant cell line RPMI8226-BTZ/100 and the RPMI8226 wild type cell line (Figure 5A and Figure S1). To confirm the involvement of GSH metabolism in overcoming bortezomib resistance, we further performed metabolomics analysis in the bortezomib-resistant cell line RPMI8226-BTZ/100. A total of 24 metabolites were differentially expressed by pretreatment compared to simultaneous treatment (Table S4). Pathway enrichment analysis with these metabolites identified purine metabolism, pyrimidine metabolism, pentose phosphate pathway, glycolysis/gluconeogenesis, alanine, aspartate and glutamate metabolism, and glutathione metabolism (enrichment *p* value = 0.0056) as the main enriched pathways (Figure 5B).

Consistent with the results in the OPM2 cell line, GSH and GSSG were decreased by pretreatment compared to the control (Figure 5C, upper panel), indicating the activation of GSH breakdown and/or decreased synthesis in pretreated cells. In addition, pyrimidine CMP, purines AMP, GMP, and their precursor IMP, as well as several purine metabolism-associated molecules, including adenine, hypoxanthine, and guanine, were highly upregulated in pretreated cells (Figure 5D), which suggested that DHA/EPA pretreatment activated the folate cycle for purine and pyrimidine synthesis in bortezomib-resistant cells. Meanwhile, SAM, the critical intermediate of the methionine cycle for cysteine synthesis, was significantly upregulated by pretreatment compared to simultaneous treatment (Figure 5D), thereby confirming the activation of methionine cycle in pretreated BTZ/100 cells. Of note, the accumulation of ribulose-5-phosphate and sedoheptulose-7-phosphate, important intermediates of the pentose phosphate pathway (PPP), was observed in pretreated BTZ/100cells (Figure 5C, lower panel), suggesting the increased activity of PPP by DHA/EPA pretreatment in these cells. A predominant function of PPP is the generation of NADPH for antioxidant defence. Therefore, the enhanced activity of PPP in pretreated cells suggests increased oxidative stress, in line with lower GSH levels. Together, these results further validated that DHA/EPA exerted synergistic toxicity when they were used before bortezomib through enhancing oxidative stress and depleting GSH levels in MM cells, especially in bortezomib-resistant cells.

### 3.6. ATF3/4 Pathway-Regulated GSH Cycle Metabolism Is Activated by DHA/EPA Pretreatment in MM Cells

**Figure 6:**
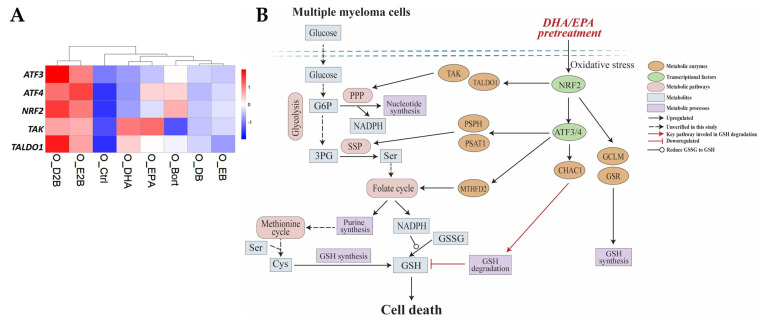
CHAC1-mediated GSH depletion may be important for bortezomib chemosensitivity in MM cells increased by DHA/EPA pretreatment. (**A**) Heatmap analysis of the expression of *ATF3*, *ATF4*, *NRF2*, *TAK*, and *TALDO1* in different conditions. (**B**) Summarizing scheme. Pretreating MM cells with DHA/EPA before bortezomib induces GSH degradation through activating the NRF2-ATF3/4-CHAC1 pathway, which eventually leads to cell death. Meanwhile, metabolic pathways of PPP, SSP, folate cycle, and methionine cycle may be activated to increase GSH synthesis to recover the cellular redox homeostasis during cell death.

Activating transcription factor 3/4 (ATF3/4), the main members of the ATF/CREB family of transcription factors that regulate the expression of a cohort of cytoprotective genes under oxidative stress, have been shown to regulate the expression of enzymes of serine synthesis and metabolism and GSH metabolism including *PSAT1*, *PSPH, MTFHD2*, *SHMT2*, and *CHAC1* [20,49]. Notably, the expression of *PSAT1*, *PSPH*, *MTFHD2*, and *SHMT2* contributes to GSH synthesis [49], while *CHAC1* functions to maintain the oxidative balance by inducing GSH degradation [20]. These specific RNA-seq results showed that the expression of all these enzymes were upregulated in pretreated OPM2 cells (Figure 4C–E). Moreover, pretreatment with DHA/EPA significantly increased *ATF3/4* compared to bortezomib treatment, whereas an opposite effect was observed by simultaneous treatment (Figure 6A).

Given the critical role of transcription factor NRF2 in activating ATF3/4 [52,53], as a next step, its transcriptional level was examined. As expected, NRF2 was highly upregulated in pretreated cells compared to bortezomib treatment, whereas it was markedly decreased by simultaneous treatment (Figure 6A), indicating the activation of NRF2-dependent pathways by DHA/EPA pretreatment in OPM2 cells. In keeping with this finding, NRF2-regulated metabolic genes, such as GCLM and GSR (GSH synthesis) and SLC7A11 (cysteine/glutamate transporter) (Figure 4C), as well as TAK and TALDO1 (PPP) (Figure 6A), were also highly expressed in pretreated cells. Taken together, our results suggested that the NRF2-ATF3/4-CHAC1 pathway-mediated antioxidant response might be associated with bortezomib cytotoxicity in MM cells increased by DHA/EPA pretreatment (Figure 6B).

## 4. Discussion

**Figure 7:**
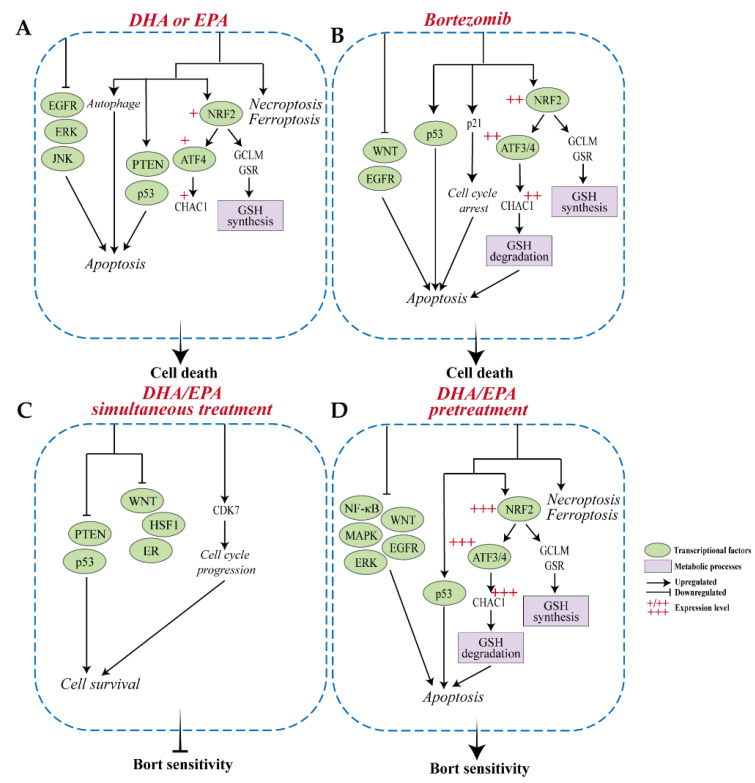
Possible mechanism underlying the opposite effects of the different treatment schedules with DHA/EPA on bortezomib chemosensitivity in MM cells. (**A**) DHA or EPA may induce MM cell death through activating PTEN and p53 signaling pathways, p62-mediated autophagy, ferroptosis, and necroptosis, and inhibiting ERK, JNK, and EGFR signaling pathways. (**B**) Bortezomib-induced MM cell death may include blocking pro-survival pathways of WNT and EGFR and activating pathways of p53 and NRF2-ATF3/4-CHAC1 and p21-mediated cell cycle arrest. (**C**) Simultaneous treatment with bortezomib and DHA or EPA may decrease bortezomib chemosensitivity in MM cells through inhibiting PTEN and p53 pathways and activating CDK7-mediated cell cycle progression. (**D**) Pretreatment with DHA or EPA prior to bortezomib increase bortezomib sensitivity possibly through activating p53 and NRF2-ATF3/4-CHAC1 pathways, ferroptosis and necroptosis and inhibiting pathways of WNT, NF-κB, MAPK, ERK, and EGFR in MM cells.

Currently, overcoming chemoresistance remains the main challenge for MM treatment. Very recently, we demonstrated that simultaneous treatment or pretreatment with DHA/EPA leads to a completely opposite effect on bortezomib chemosensitivity in MM cells [17]. In this study, by using a combined analysis of the transcriptome and metabolome of MM cells simultaneously treated or pretreated with DHA/EPA, the crucial role of GSH depletion in increasing bortezomib sensitivity in MM was investigated (Figure 7). This study provides insights for the NRF2-ATF3/4-CHAC1 pathway as a potential therapeutic target for MM. 

It is well known that the reactive oxygen species (ROS) level is consistently high in cancer cells, including MM, for cell survival. However, when its concentration reaches toxic levels, cells undergo apoptosis. Thus, cancer cells require a certain level of GSH, the major intracellular antioxidant, to maintain intracellular oxidative balance. In our study, we showed that pretreating MM cells with DHA/EPA before bortezomib potently decreased GSH levels, which tipped the oxidative balance in these cells in favor of apoptosis. In addition, GSH can be degraded by the gamma-glutamylcyclotransferase activity of CHAC1 [20], which suggests the critical role of CHAC1 in the cellular oxidative balance. Overexpression of CHAC1 in HEK 293 cells reduces GSH by degradation [20], confirming the role of CHAC1 in GSH degradation. Therefore, the increased *CHAC1* and decreased GSH/GSSG in pretreated MM cells (Figure 4B,C) indicated that the GSH degradation by DHA/EPA pretreatment may be triggered by the expression of CHAC1. The extremely low level of *CHAC1* in untreated MM cells (Figure 4C) reflected that the CHAC1 is likely to be fully eliminated under normal conditions. Therefore, the GSH degradation caused by DHA/EPA pretreatment in MM cells is conditional and can only be triggered when CHAC1 is expressed. Moreover, it has been documented that the half-life of intracellular GSH in macrophages was 1.9±0.4 h after treatment with buthionine sulfoximine [54], an inhibitor of γ-glutamylcysteine synthetase for blocking GSH synthesis. In our study, contrary to DHA/EPA, the GSH/GSSG levels were significantly decreased due to the activation of CHAC1-mediated degradation in pretreated cells (Figure 4B), although the GSH synthesis were consistently increased in both DHA/EPA-treated and pretreated cells. All of this suggests that the half-life of GSH within MM cells would be much shorter than the time of treatment (2 h pretreatment with DHA/EPA plus 4 h with bortezomib) and most of the synthesized GSH can be degraded shortly in pretreated cells.

NRF2 is a well-known transcriptional activator that functions to increase the ability of cells to adapt to oxidative stress through the upregulation of genes for anti-oxidation and cytoprotection. Additionally, NRF2 was found to redirect metabolism pathways in cancer cells to produce sufficient energy and nutrients. Pathways of pentose phosphate and GSH synthesis can be directly activated by the transcriptional activity of NRF2, while the serine synthesis pathway and folate cycle are induced by NRF2 in an ATF4-dependent manner [49]. Numerous studies have demonstrated that bortezomib executes anti-cancer activity by triggering oxidative stress-related cell death [55,56,57]. The observed higher activity of NRF2 in pretreated cells compared to bortezomib-treated cells suggests enhanced oxidative stress in these cells, thereby promoting more cell death.

Furthermore, transcriptional factor ATF4 and its target gene ATF3, another member of the ATF family of transcription factors, have been reported to regulate *CHAC1* transcription, delineating the link between oxidative status and cellular signaling pathways. Our results showed that almost equal amounts of ATF4 was increased by EPA and bortezomib, but only bortezomib potently decreased GSH, suggesting that ATF3 might play a more important role in inducing *CHAC1* transcription in MM cells. Moreover, the NRF2 target genes were highly upregulated by DHA/EPA compared to untreated cells, although NRF2 itself was only slightly increased, indicating the activation of NRF2 in these cells. However, the depletion of GSH was only observed in bortezomib-treated or DHA/EPA-pretreated cells but not in DHA/EPA-treated or simultaneously treated cells, possibly because the ATF3/4-CHAC1-GSH degradation can only be triggered when NRF2 accumulates to an adequately high level. Low levels of NRF2 in MM cells mainly leads to the upregulation of genes for GSH synthesis (*GSR* and *GCLM*). Of note, the high expression of NRF2 has been closely associated with bortezomib resistance in MM primary cells [10]. We have reported that bortezomib showed minimum toxicity in the OPM2 cell line compared to L363, U266, and MM.1S cell lines [17], suggesting that the OPM2 cell line is most resistant to bortezomib. It is interesting that the highest expression level of NRF2 was detected in the OPM2 cell line (Figure S3), potentially explaining the reason why this cell line is more resistant to bortezomib. Moreover, previous studies showed that the significantly higher level of intracellular GSH are positively correlated with bortezomib resistance in MM cells [13,50] and elevation of GSH entirely abolished bortezomib-induced cytotoxicity [51], supporting our conclusion that the cellular GSH level may be a critical factor in the determination of MM cell fate.

In addition to modulating the metabolism, ATF3 was found to play a dual role as an oncogene and tumor suppressor in multiple cancers. For example, upregulated ATF3 could be observed in prostate cancer by androgen stimulation and contributes to cell proliferation and cell cycle progression [58], suggesting the oncogenic role of ATF3. However, ATF3 also acts as a tumor suppressor in prostate cancer by blocking pro-survival pathways, such as androgen receptor and AKT pathways, ultimately leading to the inhibition of cell proliferation and invasion [59,60]. Additionally, the overexpression of ATF3 decreased the tumor size in human colon cancer xenografts and inhibited cell migration and invasion, suggesting the tumor suppressive effect of ATF3 [61]. Conversely, knockdown of ATF3 suppressed the motility and invasion of colon cancer cells [62]. Of note, the low expression of ATF3 has been implicated in bortezomib resistance and poor survival in MM patients [63]. Our result of the lowest expression level of ATF3 in untreated cells and highly increased ATF3 in DHA/EPA pretreated cells might indicate the central role of ATF3 in overcoming bortezomib resistance in MM cells.

## 5. Conclusions

By using a combination treatment of DHA/EPA and bortezomib in MM cells, the present study extends our understanding of the mechanism for bortezomib-associated resistance. Pretreating MM cells with DHA/EPA before bortezomib may increase bortezomib toxicity through orchestrating the cellular redox system. The CHAC1-mediated GSH depletion may be a main effector for triggering bortezomib-resistant MM cell death. Moreover, the expression of CHAC1 may be associated with the activity of the NRF2-ATF3/4 pathway. Thus, targeting the cellular pathways that contribute to CHAC1 expression may be a promising therapeutic strategy for overcoming bortezomib-associated resistance in MM.

## Data Availability

The datasets analyzed during the current study are available from the corresponding author on reasonable request.

## Supplementary Materials

The following are available online at https://www.mdpi.com/article/10.3390/cells10092287/s1. Figure S1: The effect of DHA/EPA on the chemosensitivity of RMPI8226 cells to bortezomib, Figure S2: The levels of CMP, adenine, hypoxanthine, and guanine upon different treatment in the BTZ/100 cell line, Figure S3: Heatmap analysis of the expression of NRF2 in four MM cell lines, Table S1: Enriched pathways identified by Reactome among DEGs in indicated clusters, Table S2: Significantly enriched pathways by Reactome of DEGs in cluster 1 of Figure 3B (*p* < 0.05), Table S3: A list of tumor suppressors and oncogenes used in all heatmaps, Table S4: A list of the metabolites for pathway enrichment analysis using MetaboAnalyst 5.0, Table S5: A list for the rest of abbreviations.
