# Supplementary material for: Omega-3 Fatty Acids DHA and EPA Reduce Bortezomib Resistance in Multiple Myeloma Cells by Promoting Glutathione Degradation
Source: Cells. 2021 Sep 2;10(9):2287. doi: 10.3390/cells10092287 (PMC8465636; doi:10.3390/cells10092287)
Supplement: Supplementary file 1 [file cells-10-02287-s001.zip › cells-1323132-supplementary.pdf]

cells MDPI

Supplementary data

Table S1 Enriched pathways identified by Reactome among DEGs in indicated clusters.

Subclusters	Top 10 most relevant pathways	*P* values	FDR	Involved genes
**Cluster 2** (Figure 1E)	Response of EIF2AK1 (HRI) to heme deficiency (R-HSA-9648895)	4.60e-12	7.78e-10	ATF3<br>CHAC1<br>DDIT3
	Cellular responses to stress (R-HSA-2262752)	6.53e-09	4.59e-07	ATF3<br>CHAC1<br>DDIT3<br>CDKN1A<br>DEDD2
	Cellular responses to external stimuli (R-HSA-8953897)	8.20e-09	4.59e-07	ATF3<br>CHAC1<br>DDIT3<br>CDKN1A<br>DEDD2
	Transcriptional activation of p53 responsive genes (R-HSA-69560)	1.08e-07	3.56e-06	CDKN1A
	Transcriptional activation of cell cycle inhibitor p21 (R-HSA-69895)	1.08e-07	3.56e-06	CDKN1A
	ATF4 activates genes in response to endoplasmic reticulum stress (R-HSA380994)	2.13e-07	5.95e-06	ATF3<br>DDIT3
	Transcriptional Regulation by TP53 (R-HSA-3700989)	3.35e-07	8.05e-06	ATF3<br>DDIT3<br>CDKN1A<br>PMAIP1
	PERK regulates gene expression (R-HSA-381042)	4.91e-07	1.03e-05	ATF3<br>DDIT3
	Response of EIF2AK4 (GCN2) to amino acid deficiency (R-HSA-9633012)	7.33e-07	1.32e-05	ATF3<br>DDIT3
	TP53 Regulates Transcription of Genes Involved in G1 Cell Cycle Arrest (RHSA-6804116)	3.94e-06	6.31e-05	CDKN1A
**Cluster 3 and 4** (Figure 1E)	TP53 Regulates Metabolic Genes (R-HSA-5628897)	1.36e-04	0.006	PTEN
	Regulation of PTEN mRNA translation (R-HSA-8943723)	2.60e-04	0.006	PTEN
	PTEN Regulation (R-HSA-6807070)	3.32e-04	0.006	PTEN
	Regulation of PTEN gene transcription (R-HSA-8943724)	0.001	0.017	PTEN
	PIP3 activates AKT signaling (R-HSA-1257604)	0.002	0.017	PTEN
	Iron uptake and transport (R-HSA-917937)	0.002	0.017	FTH1
	PTEN Loss of Function in Cancer (R-HSA-5674404)	0.002	0.017	PTEN
	Intracellular signaling by second messengers (R-HSA-9006925)	0.003	0.018	PTEN
	Transcriptional Regulation by MECP2 (R-HSA-8986944)	0.003	0.018	PTEN
	Transcriptional Regulation by TP53 (R-HSA-3700989)	0.007	0.033	PTEN
**Cluster 1** (top 5 genes, Figure 2E)	Regulation of HSF1-mediated heat shock response (R-HSA-3371453)	3.50e-09	6.34e-07	DNAJB1<br>HSPH1<br>DEDD2
	Cellular response to heat stress (R-HSA-3371556)	8.48e-09	7.63e-07	DNAJB1<br>HSPH1<br>DEDD2
	Attenuation phase (R-HSA-3371568)	1.36e-08	8.19e-07	DNAJB1<br>HSPH1<br>DEDD2

	HSF1-dependent transactivation (R-HSA-3371571)	3.38e-08	1.52e-06	DNAJB1<br>HSPH1<br>DEDD2
	HSF1 activation (R-HSA-3371511)	2.15e-06	5.50e-05	HSPH1<br>DEDD2
	Cellular responses to stress (R-HSA-2262752)	2.20e-06	5.50e-05	DNAJB1<br>HSPH1<br>DEDD2<br>PSME1
	PTEN Regulation (R-HSA-6807070)	2.31e-06	5.50e-05	PSME1<br>PTEN
	Cellular responses to external stimuli (R-HSA-8953897)	2.50e-06	5.50e-05	DNAJB1<br>HSPH1<br>DEDD2<br>PSME1
	PIP3 activates AKT signaling (R-HSA-1257604)	2.69e-05	5.37e-04	PSME1<br>PTEN
	Intracellular signaling by second messengers (R-HSA-9006925)	4.59e-05	8.27e-04	PSME1<br>PTEN

FDR: False discovery rate; R-HSA-XXXXXXX: Reactome pathway identifiers

Table S2 Significantly enriched pathways by Reactome of DEGs in cluster 1 of Figure 3B ($p < 0.05$).

**Significant pathways**	***P* values**	**FDR**	**Involved genes**
Toxicity of botulinum toxin type G (botG) (R-HSA-5250989)	0.005	0.06	SYT1
Cargo recognition for clathrin-mediated endocytosis (R-HSA-8856825)	0.007	0.06	SYT1
Protein repair (R-HSA-5676934)	0.007	0.06	MSRA
Signaling by MAPK mutants (R-HSA-9652817)	0.008	0.06	DUSP8
Toxicity of botulinum toxin type B (botB) (R-HSA-5250958)	0.01	0.06	SYT1
Clathrin-mediated endocytosis (R-HSA-8856828)	0.013	0.06	SYT1
Acetylcholine Neurotransmitter Release Cycle (R-HSA-264642)	0.02	0.06	SYT1
Serotonin Neurotransmitter Release Cycle (R-HSA-181429)	0.022	0.06	SYT1
Norepinephrine Neurotransmitter Release Cycle (R-HSA-181430)	0.022	0.06	SYT1
GABA synthesis, release, reuptake and degradation (R-HSA-888590)	0.023	0.06	SYT1
Neurotoxicity of clostridium toxins (R-HSA-168799)	0.026	0.06	SYT1
Dopamine Neurotransmitter Release Cycle (R-HSA-212676)	0.027	0.06	SYT1
Glutamate Neurotransmitter Release Cycle (R-HSA-210500)	0.029	0.06	SYT1
RAF-independent MAPK1/3 activation (R-HSA-112409)	0.029	0.06	DUSP8
TNFR1-induced NF-kappa B signaling pathway (R-HSA-5357956)	0.036	0.06	RNF31
EGFR downregulation (R-HSA-182971)	0.037	0.06	PTPRK
Membrane Trafficking (R-HSA-199991)	0.038	0.06	SYT1
Gluconeogenesis (R-HSA-70263)	0.04	0.06	SLC37A1
Ovarian tumor domain proteases (R-HSA-5689896)	0.045	0.06	OTUB2
Regulation of TNFR1 signaling (R-HSA-5357905)	0.046	0.06	RNF31

FDR: False discovery rate; R-HSA-XXXXXXX: Reactome pathway identifiers

Table S3 A list of tumor suppressors and oncogenes used in all heatmaps.

**Tumor suppressors**	**Tumor suppressors**	**Oncogenes**	**Oncogenes**	**Oncogenes**
NKD2	*FTH1*	*FZD8*	*TSC22D3*	*HMOX2*
GAS1	*NQO1*	*POU3F2*	*CDK7*	*BOLA2B*
PTPRK	*PSME1*	*FLII*	*JDP2*	*BFAR*
PINX1	*DNAJB1*	*NRG2*	*OTUB2*	*KDM7A*
DDIT4	*HSPH1*	*LDLRAD4*	*HRAS*	*LUCAT1*
GABARAPL1	*MIR1244-3*	*KLF2*	*PEAK3*	*CHORDC1*
SQSTM1	*MTCH2*	*PTPRK*	*PHF1*	*TXNRD1*
DUSP8	*IBTK*	*MAP3K19*	*RUFY1*	*HMOX1*
DEDD2	*RASA3*	*SLC25A24*	*CDR2*	*SRXN1*
PAK1IP1	*RNF144A*	*HSPA4L*	*RRN3*	*PAK1IP1*
ATF3	*FRG1*	*HSP90AA1*	*SLC37A1*	
PMAIP1	*NMRAL1*	*DNAJA1*	*MSRA*	
DDIT3	*NINJ1*	*HSPA6*	*DUSP8*	
CHAC1	*TRIB3*	*BAG3*	*RNF31*	
MXD1	*LAIR1*	*SERPINH1*	*SYT1*	
DNAJB4	*PTEN*	*ZFAND2A*	*NANOS1*	
CDKN1A	*EID3*	*ZNF251*	*BCL6*	
INHBE	*OSGIN1*	*CLU*	*CYP19A1*	
IFI27L1	*IFI27L2*	*WNT11*	*EPHB1*	

PAK1IP1: play a dual role as an oncogene and tumor suppressor in cancer cells.

Table S4 A list of the metabolites for pathway enrichment analysis using MetaboAnalyst 5.0.

**Metabolites-OPM2** (Fig. 4A)		**Metabolites-BTZ/100** (Fig. 5B)		
HexP	ASN	HexP	Dihydroxyacetone3P	Hypoxanthine
AMP	Glutathione	AMP	3-Hydroxybutanoic Acid(BHBA)	Fructose 16bisP
GMP	GLN	GMP	Ribulose5P	PEP
IMP	NADP+	IMP	CMP	Adenine
GSSG	NADPH	GSSG	SER	GLN
S–Adenosyl–Methionine	Ornithine	S–Adenosyl–Methionine	Sedoheptulose7P	Guanine
NADH	Glucose	ASN	Ornithine	Orotate
LYS	Orotate	Glutathione	Glucose	N-Carbamoyl-aspartate

Table S5 A list for the rest of abbreviations

**Abbr.**	**Description**	**Abbr.**	**Description**
AKT	Protein kinase B (PKB)	IMP	Inosine monophosphate
AMP	Adenosine monophosphate	JAK2	Janus kinase 2
CDK7	Cyclin-dependent kinase 7	JNK	C-Jun N-terminal kinase
CMP	Cytidine monophosphate	KLF2	Kruppel Like Factor 2
CREB	CAMP response element-binding protein	MAPK	Mitogen-activated protein kinase
EGFR	Epidermal growth factor receptor	MEK1/2	Mitogen-activated protein kinase kinase 1/2
ER	Estrogen receptor	MKK7	MAP-kinase kinase 7
ERBB	Erb-B2 Receptor Tyrosine Kinase	NADPH	Nicotinamide adenine dinucleotide phosphate
ERK	Extracellular-signal-regulated kinase	NF-κB	Nuclear factor-kappa B
GCLM	Glutamate-Cysteine Ligase Modifier Subunit	PI3K	Phosphatidylinositol-3-kinase
GMP	Guanosine monophosphate	PTEN	Phosphatase and tensin homolog
GSR	Glutathione-Disulfide Reductase	STAT3	Signal transducer and activator of transcription 3
HER	(also termed ERBB)	TNFR1	Tumor Necrosis Factor Receptor 1
HSF-1	Heat Shock Transcription Factor-1	WNT	Wingless/Integrated
IKKβ	Inhibitor of NF-κB kinase subunit beta		

Figure S1

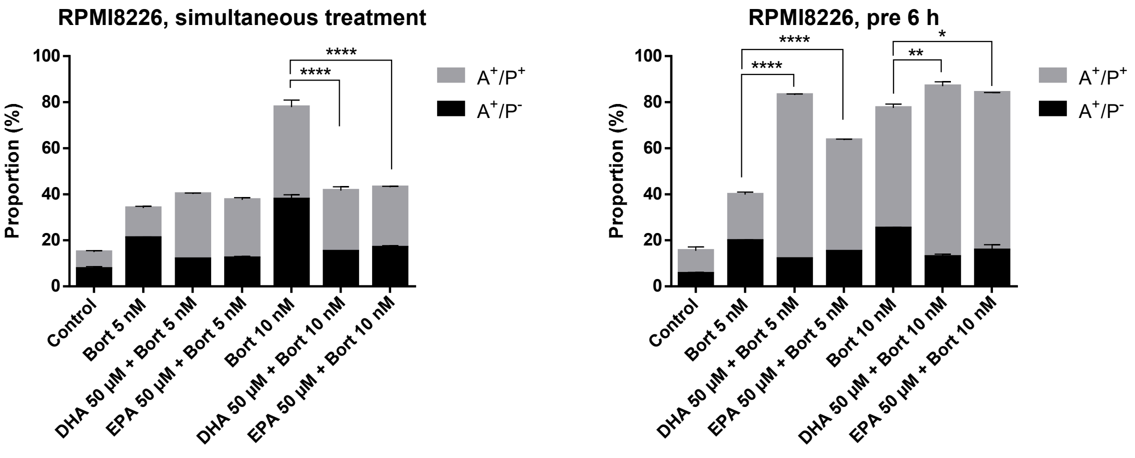

Figure S1 The effect of DHA/EPA on the chemosensitivity of RMPI8226 cells to bortezomib. RPMI8226 cells were pretreated with 50 μM of DHA/EPA for 0 or 6 h and then incubated with bortezomib (5 or 10 nM) for 24 h. Apoptotic cells were determined by Annexin V and PI staining. Data are presented as mean ± SD of two treatments. *$p < 0.05$, **$p < 0.01$, ****$P < 0.0001$.

Figure S2

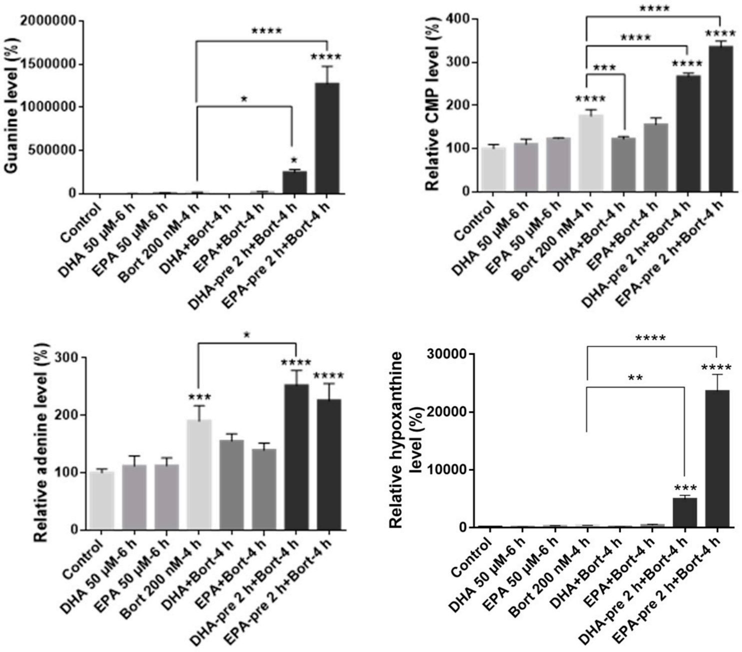

Figure S2 The levels of CMP, adenine, hypoxanthine, and guanine upon different treatment in BTZ/100 cell line. RPMI8226-BTZ/100 was pretreated with 50 μM of DHA or EPA for 0 or 2 h and then incubated with bortezomib (200 nM) for 6 h. The levels of CMP, adenine, hypoxanthine, and guanine were determined by metabolomics analysis. Data are presented as mean ± SD of three independent treatments. ** $p < 0.01$, *** $p < 0.001$, **** $p < 0.0001$ when compared with control.

Figure S3

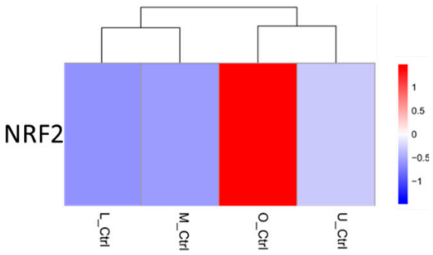

Figure S3 Heatmap analysis of the expression of *NRF2* in four human MM cell lines L363 (L), MM.1S (M), OPM2 (O) and U266 (U).
